# Cultures of Practice: Specialty-Specific Differences in End-of-Life Conversations

**DOI:** 10.1089/pmr.2020.0054

**Published:** 2021-03-24

**Authors:** Andre Morales, Kevan C. Schultz, Shasha Gao, Alan Murphy, Amber E. Barnato, Joseph B. Fanning, Daniel E. Hall

**Affiliations:** ^1^ Department of Medicine, Center for Biomedical Ethics and Society, Vanderbilt University Medical Center, Nashville, Tennessee, USA.; ^2^ University Center for Social and Urban Research (UCSUR), University of Pittsburgh, Pittsburgh, Pennsylvania, USA.; ^3^ Center for Health Equity Research and Promotion, VA Pittsburgh Healthcare System, Pittsburgh, Pennsylvania, USA.; ^4^ OhioHealth, Columbus, Ohio, USA.; ^5^ Department of Medicine, The Dartmouth Institute for Health Policy and Clinical Practice, Geisel School of Medicine at Dartmouth, Lebanon, New Hampshire, USA.; ^6^ Department of Surgery, VA Pittsburgh Healthcare System, Pittsburgh, Pennsylvania, USA.; ^7^ Department of Surgery, University of Pittsburgh, Pittsburgh, Pennsylvania, USA.

**Keywords:** communication, goals of care, medical specialty, patient preference

## Abstract

***Importance:*** Goals of care discussions at the end of life give opportunity to affirm the autonomy and humanity of dying patients. Best practices exist for communication around goals of care, but there is no research on differences in approach taken by different specialties engaging these conversations.

***Objective:*** To describe the communication practices of internal medicine (IM), emergency medicine (EM), and critical care (CC) physicians in a high-fidelity simulation of a terminally ill patient with stable and defined end-of-life preferences.

***Design, Setting, and Participants:*** Mixed-methods secondary analysis of transcripts obtained from a multicenter study simulating high stakes, time-limited end-of-life decision making in a cohort of 88 volunteer physicians (27 IM, 22 EM, and 39 CC) who were called to evaluate a standardized patient in extremis. The patient had clear comfort-oriented goals of care that the physician needed to elicit and use to inform treatment decisions. Discussions were coded at the level of the sentence for semantic content.

***Exposures:*** Data were analyzed by physician specialty.

***Main Outcome Measure:*** Occurrence of content codes indicative of prudent (right outcome by the right means) goals of care conversations. Data were analyzed both for number of occurrences of the code in a simulated conversation and for presence or absence of the code within a conversation.

***Results:*** There was no difference between physician types in intubation rates or intensive care unit admissions. Codes for “comfort as a goal of care,” “noncurative goals of care,” and “oblique references to death” emerged as significantly different between physician types.

***Conclusions and Relevance:*** This experiment shows demonstrable differences in practice patterns between physician specialties when addressing end-of-life decision making. Some of the variation likely arose from differences in setting, but these data suggest that training in goals of care conversations may benefit if it is adapted to the distinct needs and culture of each specialty.

## Introduction

Patients should not be subjected to treatments that violate their settled informed preferences. As a truism of patient care, all health care professionals—no matter the specialty—are expected to endorse this ethical commitment.^1^ To uphold this obligation, care teams, patients, and their health care decision makers must communicate to achieve these preferences. When receiving care at the end of life, patients are not always treated in line with their preferences.^2^ Despite the widespread need for serious illness conversations to guide these decisions, most physicians report inadequate training in this area of practice^3^ and likely develop varied communication skills influenced by their culture of practice. Recognizing this trend, the medical literature is beginning to identify best practices for discussion of goals at critical junctures,^3^ and with better understanding of current communication approaches their standards could be more effectively taught across medical specialties.

Certain skills are commonly acknowledged as good practice when conducting end-of-life conversations. Physicians must have an accurate understanding of the patient's prognosis and be able to convey this prognosis effectively.^4^ Some resources advocate using direct language, such as “cancer” or “death,” in the spirit of speaking frankly and clearly.^5^ Providers should anticipate that patients will have an emotional reaction to this information and attend to these emotional cues, acknowledging their presence and attending to the patient's social needs before proceeding to the next step in defining care goals.^6^ Physicians must ascertain the values of the patient, and may choose to summarize these goals to confirm alignment with the patient's needs.^7^ Expert physicians are noted to devote proportionally more time to build partnership with the patients and allow for patient expression of opinion, understanding, and beliefs.^8^

In executing these best practice guidelines, clinicians encounter challenges specific to their practice environments. For example, physicians who do not have long-term relationships with their patients must invest extra time in building a therapeutic alliance with the patient or must develop strategies to efficiently promote trust and openness. Clinicians who will not be the final care provider must determine how much of the conversation is important to have in their setting, and what should be explored with the long-term provider.^9^ Some clinicians encounter patients who cannot express their own wishes and must, therefore, interact with health care proxies to determine the course of action most in line with those patients' wishes.^10^ We posit that, with time, characteristics unique to the setting of practice for different specialty types produce variation in the practice of serious illness conversation, which then creates a culture that further informs the diverging norms of practice for different specialties.

Prior study demonstrates formation of discrete cultures early in medical training, and also evidence that specialty-specific differences in care exist. For example, research in the areas of anthropology, sociology, and social psychology has addressed the ways a culture can shape its participants.^11–16^ Perceived differences between medical specialties arise before and during training, reinforcing stereotypes that may encompass both the positive and negative traits of the typical practitioner of the various types of medicine.^17–19^ Differences in treatment styles have been demonstrated when different specialties manage the same illness, such as mental health^16^ or coronary artery disease.^14^ Practice characteristics differ in areas such as patient-centeredness^12^ and the aspects of communication that are important for patient satisfaction.^15^

This study aims to discover and characterize specialty-specific differences in end-of-life communication, focusing on care plans that aim at the right end (goal concordant) using the right means (preference concordant) and thus meet criteria for what virtue* theory would consider “prudent” practice.^20–23^ Given the importance of conversations regarding end-of-life care, it is crucial to understand the specialty-specific cultures that guide these practices. We focus on practitioners from three specialties—internal medicine (IM), critical care (CC) medicine, and emergency medicine (EM)—to evaluate their practice during a simulated, time-constrained goals of care discussion with a dying patient, to identify differences in communication that may reflect the cultures embedded in the specialties.

## Methods

This study is a mixed-methods secondary analysis of transcripts obtained from a multicenter study simulating high stakes, time-limited end-of-life decision making. All procedures were approved by the University of Pittsburgh Institutional Review Board. The details of the primary simulation and its validation have been previously published.^24^ The simulation prompts physician volunteers from the departments of IM, CC, and EM to evaluate and treat an elderly male with symptoms suggestive of impending cardiopulmonary arrest. The patient's chart reveals a diagnosis of widely metastatic cancer, and that his symptoms are most likely due to progression of his underlying terminal disease. He is accompanied by his health care surrogate. Although there is no documentation of code status or advance care planning, the patient and his surrogate have discussed the patient's preferences for palliation as opposed to escalating care due to his recent unpleasant experience in the intensive care unit (ICU). The patient is awake but dyspneic for most of the encounter, with worsening physiological measurements that compel the treating physician to choose either intubation or comfort care. The encounters were recorded and transcribed, providing the material for our secondary analysis, and the ultimate outcome of each simulation was known with regard to intubation, transfer to the ICU, and orders specifying comfort measures only (CMO) or “do not resuscitate/do no intubate” (DNR/DNI).

The secondary analysis of these simulated patient encounters was conducted in two parts: A codebook was developed to qualitatively and quantitatively explore the elements of “prudent” care identified in the transcripts and these quantified elements were then qualitatively reanalyzed through thematic analysis. The concept of prudence, in this case, arose from the Aristotelian concept of *phronesis*, defined as the virtue required to achieve the right end (e.g., a comfortable dying process) through the right means (e.g., patient-centered decision making).^20^ This secondary analysis was an effort to explore the empirical, rather than simply theoretical, identification of prudence in (simulated) clinical practice and to show that it could be defined, identified, and analyzed to show clinically meaningful differences in care. Our goal was to apply a novel method of study for complex behaviors that could rigorously identify these behaviors and also provide a platform for a deductive exploration of their use. In particular, we sought to determine whether the differences in specialty practices might lead to observably different patterns of prudent behavior, adapted to the needs of the particular specialty, but not necessarily transferrable to contexts outside the central focus of that specialty. Further explanation of the methodological and philosophical rationale for this analysis are described elsewhere.^21–23,25^

First, a mixed-methods qualitative and quantitative analysis was performed to identify patterns of behavior consistent with the philosophical virtue of prudence. The authors identified a set of actions and semantic content that could be used to identify actions that would help or hinder prudent care on the part of the treating clinician, using theoretical understanding of prudence without reference to the transcripts and then iteratively applying the codes to the encounters to refine the codebook. The final codebook included 6 actor codes (e.g., patient, physician, and nurse), 13 action codes (e.g., asking a question, challenging, telling information, and recommending), and 61 content codes (e.g., prognostic information, diagnostic information, code status, and goals of care). Primary coding of the transcripts was completed using Atlas.ti (Version 7, Germany) by trained analysts working in pairs with adjudication by the lead analyst (K.S.), as necessary. The unit of coding was the sentence, and coding was adjudicated until consensus was reached for each unit. After primary coding, the authors (D.E.H., K.S., and J.B.F.) used Atlas.ti functionality to build 12 “supercodes” comprising specific actor, action, and content codes to identify text that exhibited specific kinds of behavior (e.g., “Physician names death” is a supercode consisting of the “physician” actor code and either of the two content codes pertaining to a “direct” or “oblique” reference to death).

Quantitative analysis of qualitative coding began by exporting the final adjudicated database from Atlas.ti in a format suitable for statistical analysis. We tabulated counts for each code and supercode at the level of the physician (e.g., for each physician's simulation, we tabulated the total number of quotations containing each code or supercode). We then calculated mean counts for each code and supercode for each physician type (e.g., CC, EM, and IM hospitalist), testing differences between means across the three types of physicians using analysis of variance (parametric) and Kruskal–Wallis (nonparamentric) tests with the hypothesis that there would be differences in the presence of prudence-related codes. For those codes exhibiting significant differences across physician type, we tested pairwise differences with Tukey's studentized range test for value with significance at *p* < 0.05. We then created a binary indicator variable for each code and supercode to indicate if it was ever applied to a sentence from each physician's simulation (e.g., if a physician never mentioned “comfort as a goal of care,” this indicator variable was assigned the value “0,” and regardless of the number of times the physician mentioned “comfort as a goal of care,” the indicator variable was assigned the value “1” so long as the code was applied at least 1 time during the transcript). Differences between physician types were compared with chi-squared tests. Pairwise comparisons were made using Fisher's exact test, adjusting significance with a Bonferroni correction to *p* = 0.017. Finally, we constructed a series of regression models to examine the association of coded behavior with both the outcomes of interest (e.g., intubation, DNR/DNI orders, and disposition) and the physician type.

Findings from the quantitative analysis informed the second stage of inquiry, which comprised a thematic analysis exploring the qualitative characteristics of the codes identified in the prior analysis to demonstrate significant differences in use between specialty types. The authors (J.B.F. and A.M.) analyzed latent themes to understand the use and meaning of the codes that could explain their differential use between the physician types.^26^ J.B.F. is an established investigator with qualitative expertise and an interest in communication skills and development of therapeutic alliances; A.M. has experience in clinical medicine and training in qualitative methods of research. These authors both read the transcripts for familiarity; A.M. then generated initial themes based on the codes that had been identified as distinct between groups in the qualitative phase. A.M. and J.B.F. then refined these themes, and conflicts were discussed until resolution was reached. A.M. and J.B.F. then reviewed the identified themes to confirm consistency with the dataset before A.M. named and refined the themes. The authors examined the context [e.g. circumstances and consequences] of each coded unit, then developed categories of use and elucidated the multiplicity of meanings represented by the units to describe a paradigm for communication operative in the transcripts that demonstrated prudent decision making. These categories were compared across physician types, highlighting areas of commonality and divergence to conceptualize the subtle textual differences between each physician group.

## Results

The final dataset included transcripts from 88 simulations with 27 IM, 22 EM, and 39 CC physicians (demographic characteristics stratified by specialty type available in Supplementary Material). The dataset comprised >19,000 individual sentences, each coded according to the scheme described earlier. As noted earlier, 6 agent codes, 13 action codes, 61 content codes, and 12 supercodes were identified and analyzed for patient outcome and for comparison between specialties; details regarding the complete coding scheme are described elsewhere.^25^ Three codes demonstrated significant difference between physician types (Table 1): “comfort” as a goal of care (K22), noncurative goals of care (K59), and oblique references to death (K41). These codes revealed a pattern by which IM-trained physicians were more likely to reference death and “comfort” than CC physicians, and in turn the CC physicians were more likely to reference these concepts than the EM-trained physicians. However, pairwise differences were only significant between EM and CC or EM and IM. There were no significant pairwise differences between IM and CC. A similar pattern was found with two supercodes specific to physicians recommending preferences, values, and goals of care (SC12 and SC31), suggesting that IM physicians are the most directive and EM physicians the least directive. Not surprisingly, CC physicians were more likely to reference the ICU than their EM or IM counterparts (K87).

**Table 1.** Differences between Physician Types Comparing Mean within Simulation Code Counts

Code		CC (*N* = 39)		EM (*N* = 22)		IM (*N* = 27)		*p*	*p*
		Mean	Δ	Mean	Δ	Mean	Δ	ANOVA	KW
Comfort as the goal of care	K22	5.4	ND	2.5	IM	8.3	EM	0.0005	0.0015^***^
Oblique discussion of death	K41	1.9	EM	1.5	IM CC	3.8	EM	0.0119	0.0028^***^
Physician's goal: oblique death	K53	0.2	ND	0.2	ND	0.7	ND	0.0270	0.0361^**^
Comfort/noncurative/allow-to-die	K59	2.5	ND	1.5	IM	4.3	EM	0.0477	0.0339^**^
Physician recommends preferences, values, or goals	SC12	3.6	ND	2.5	IM	5.3	EM	0.0335	0.0532^*^
Physician tells *patient* preferences	SC31	3.9	ND	1.7	IM	6.1	EM	0.0010	0.0028^*^

Δ Pairwise differences were compared using Tukey's studentized range test for value with significance at *p* < 0.05. The physician types with which there are differences are indicated as CC, EM, IM, or ND. *Note:* Combination codes were examined carefully to ensure that the statistical significance was strengthened due to the combination. For example, comfort as the goal of care was so significant that when combined with “direct death,” the combination was also significant, but the *p*-value increased from the “comfort” alone. For that reason, only the combinations that demonstrated strengthened statistical significance are reported.

^*^ *p* < 0.10 (trend only).

^**^ *p* < 0.05.

^***^ *p* < 0.01.

ANOVA, analysis of variance; CC, critical care; EM, emergency medicine; IM, internal medicine; KW, Kruskal–Wallis; ND, no difference.

When the occurrence of comfort as a goal of care (K22) is dichotomized to present/absent, there is no difference between physician types (Table 2). IM physicians referenced comfort more frequently than CC practitioners who in turn referenced it more frequently than EM physicians, but the differences were not significant. There was convergence between the binary and mean-count data regarding the more frequent presence of noncurative goals of care (K59) and oblique references to death (K41) in IM physician simulations compared with the other two physician groups.

**Table 2.** Differences between Physician Types (Comparison of Binary Data)

Code		CC (*N* = 39)		EM (*N* = 22)		IM (*N* = 27)		*p*
		*n* (%)	Δ	*n* (%)	Δ	*n* (%)	Δ	
Comfort as the goal of care	K22	31 (79)	ND	14 (64)	ND	23 (85)	ND	0.2154^NS^
Comfort and oblique discussion of death	K26	10 (26)	EM	1 (5)	IM EM	12 (44)	EM	0.0045^**^
Oblique discussion of death	K41	27 (69)	ND	7 (32)	IM	21 (78)	EM	0.0054^**^
Patient's goal: comfort/noncurative/allow-to-die	K48	10 (26)	IM	5 (23)	IM	15 (56)	EM CC	0.0227^*^
Physician's goal: comfort/noncurative/allow-to-die	K52	16 (41)	IM	10 (45)	IM	22 (81)	EM CC	0.0028^**^
Comfort/noncurative/allow-to-die	K59	20 (51)	IM	13 (59)	ND	22 (81)	CC	0.0383^*^

*Note:* Pairwise comparisons between physician types were made using Fisher's exact tests and findings are indicated in the Δ column to show ND or the physician type with which there is a difference. *Note:* Combination codes were examined carefully to ensure that the statistical significance was strengthened due to the combination. For example, combining comfort/noncurative/allow-to-die with patient actor or physician actor drives the *p*-value down from 0.038 to 0.023 and 0.003, respectively. Combinations without a strengthened statistical significance are not reported. Tests for difference across the three physician types are computed using Fisher's exact test.

^*^ *p* < 0.05.

^**^ *p* < 0.01.

NS Not statistically significant.

Multivariable models predicting outcomes by physician type showed no differences for intubation, but both IM and CC physicians were more likely than EM physicians to order CMO (odds ratio [OR] 6.33, 95% confidence interval [CI] 1.81–22.11 and OR 3.11, 95% CI 1.01–9.63, respectively). There were no differences between EM physicians and either CC or IM physicians with regard to DNR/DNI orders or transfer to the ICU. However, CC physicians were more likely than IM physicians to transfer the patient to the ICU (OR 3.074, CI 1.086–8.71) and less likely to change the code status to DNR/DNI (OR 0.25, CI 0.063–0.968).

### Qualitative analysis

Our quantitative analysis revealed three codes regarding physician–patient interactions that varied between specialties, and thus became the focus of the thematic analysis: comfort as a goal of care (K22), noncurative goals of care (K59), and oblique references to death (K41). The quantitative analysis had demonstrated a significant difference in the frequency of use of these codes; we sought to further characterize their context and semantic use with an emphasis on difference between specialties. Thematic analyses of these codes were as follows.

### Comfort as a goal of care (K22)

#### Description of the code

**FIG. 1.**
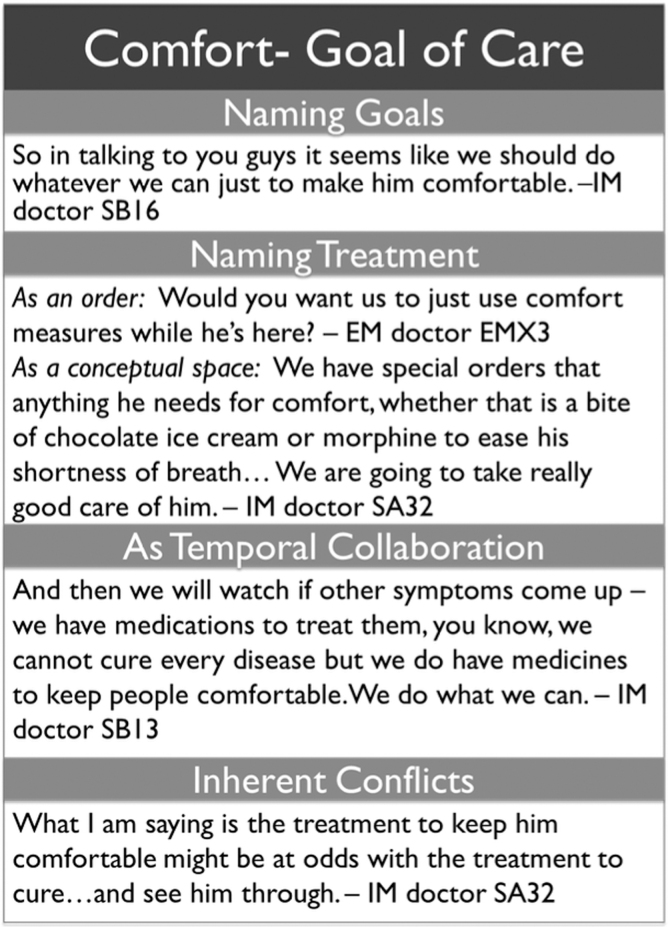
Qualitative patterns in the use of “comfort” as a goal of care. EM, emergency medicine; IM, internal medicine.

Code K22 was applied to text referring to the use of the term “comfort” in describing goals of end-of-life care. Readers flagged the word “comfort” and then distinguished uses related to goals of care from other uses of the word, such as reactions to treatment or descriptions of the patient's emotional state. Analyzing the use of K22 in the simulated encounters revealed the many possible uses of “comfort” in describing care. “Comfort” was used to name goals, delineate treatment actions, and describe the process of noncurative treatment (Fig. 1).

#### Naming goals

Physicians often specified “comfort” after hearing requests for “no tube” and “keep him comfortable” from the family, translating a specific patient preference into a more comprehensive and actionable goal of care that could be used to direct therapy beyond specific treatment requests. The word “comfort” was used to describe multiple types of goals. Most represented was symptom management: physicians observed or suggested a physiological symptom, such as shortness of breath or pain, and then pointed out that comfort-oriented care's primary aim was to reduce the burden of these symptoms: “One of the things that we can do is start morphine to help him feel a little bit more comfortable and it also reduces the sensation of shortness of breath” (Participant 1–1).

#### Comfort as a treatment plan

A second category of usage referred to the plan of action the physician would take on the patient's behalf. Most common was the use of “comfort” as the foil to invasive care; “focusing on comfort” was offered as the alternate option to intubation or chest compressions: “But, if he doesn't want to be intubated, our best job right now is to keep him comfortable, okay?” (Participant 2–25). “Comfort” as a plan of action was used in two ways—either as a singular intervention or as an iterative process from which treatments could be identified as needs changed. In the first use, “comfort” was discussed as the intervention that would be provided to the patient, much like administration of a medication or performance of a procedure: “Would you want us to just use comfort measures while he is here?” (Participant EMX3). In the latter use, physicians who had elicited comfort as a goal of care often emphasized that this treatment would require continual communication between the patient and his medical team. For these physicians, “comfort” included a temporal element in which the specific treatment actions would vary with the changing clinical picture: “And then we will watch if other symptoms come up we have medications to treat them, you know, we cannot cure every disease but we do have medicines to make people comfortable. We do what we can” (Participant 1–13). Finally, relationships were occasionally the focus of a “comfort” intervention: physicians would note that the patient's well-being would be improved by the presence of loved ones or spiritual support, and would suggest contacting these supporters as a means of comforting the patient: “Let us focus on you two spending some time together now that he is more comfortable” (Participant 1–13).

#### Conflicts of comfort

Some physicians found it important to highlight the conflicts inherent in pursuing comfort. A majority of physicians who referred to comfort care expounded the benefits, such as symptom control and peace during the dying process. Some physicians, however, emphasized that comfort care could accelerate the dying process, especially when explaining the effects of morphine on breathing: “With the medications that would make him more comfortable, it could potentially, actually, slow down his breathing, and he could be in a position where he would pass away a little faster” (Participant 2–14). No physician explicitly invoked the doctrine of double effect, which in some instances justifies the foreseen but unintended adverse effects of a treatment if the treatment's intended effect is morally acceptable, but the physicians who highlighted the potentially life-shortening consequences of comfort measures made it a point to balance the conversation of desired effects with the unintended consequences.

#### Comparison of specialties

The quantitative data reflecting use of “comfort” in describing goals of care showed that all three specialties were equally likely to incorporate this idea in the patient encounters, but that IM physicians had more instances of the code than their EM colleagues. This finding likely relates to the richness of IM's use of the word “comfort”; IM physicians typically harnessed several of the meanings described earlier. IM doctors were generally more expansive in their use, with more attention given to explaining the process of comfort care. Particularly overrepresented in IM conversations were the relational aspects of comfort and the balanced conversations about the benefits and drawbacks of comfort care. In contrast, EM physicians, who quantitatively used “comfort” fewer times, typically used “comfort” to gather and categorize family preferences, and often presented “comfort” as a single intervention rather than an iterative process.

### Noncurative goals of care (K59)

#### Description of the code

Another code that showed significant differences in use between physician groups was the delineation of explicitly noncurative goals of care (K59). Statements that addressed plans of care could be placed in the mutually exclusive categories of “noncurative,” “curative,” and “intermediate” based on content and context. The same statements were also coded for referring to physician goals or patient/surrogate goals.

For clarity, this “noncurative goal of care” code contains significant overlap with the aforementioned code “Comfort—Goal of Care” in that most mentions of comfort were in the context of noncurative goals of care. This was an intentional, *a priori* aspect of the codebook, since physicians may use “comfort” as a euphemism that fails to convey the full meaning of noncure. For example, “comfort” was also used in the context of “intermediate” treatment goals, and thus comfort was not always synonymous with “noncurative” goals of care. Goals of care were coded as noncurative only after it became clear that curative options were being eliminated. In the study design, the patient and surrogate were trained to express his desire “to be comfortable”; however, this statement was not coded as a noncurative goal of care until the physician, patient, or surrogate expressed a desire for comfort that was incompatible with curative goals.

As described earlier, this code carried significant semantic overlap with statements of comfort as a goal of care. Its semantic content was nearly equivalent: themes of delineating care goals, defining treatment options, and respecting autonomy again arose when physicians expressed noncurative goals of care. However, additional insight can be gained from the contextual paradigm of its use.

#### The noncurative paradigm

**FIG. 2.**
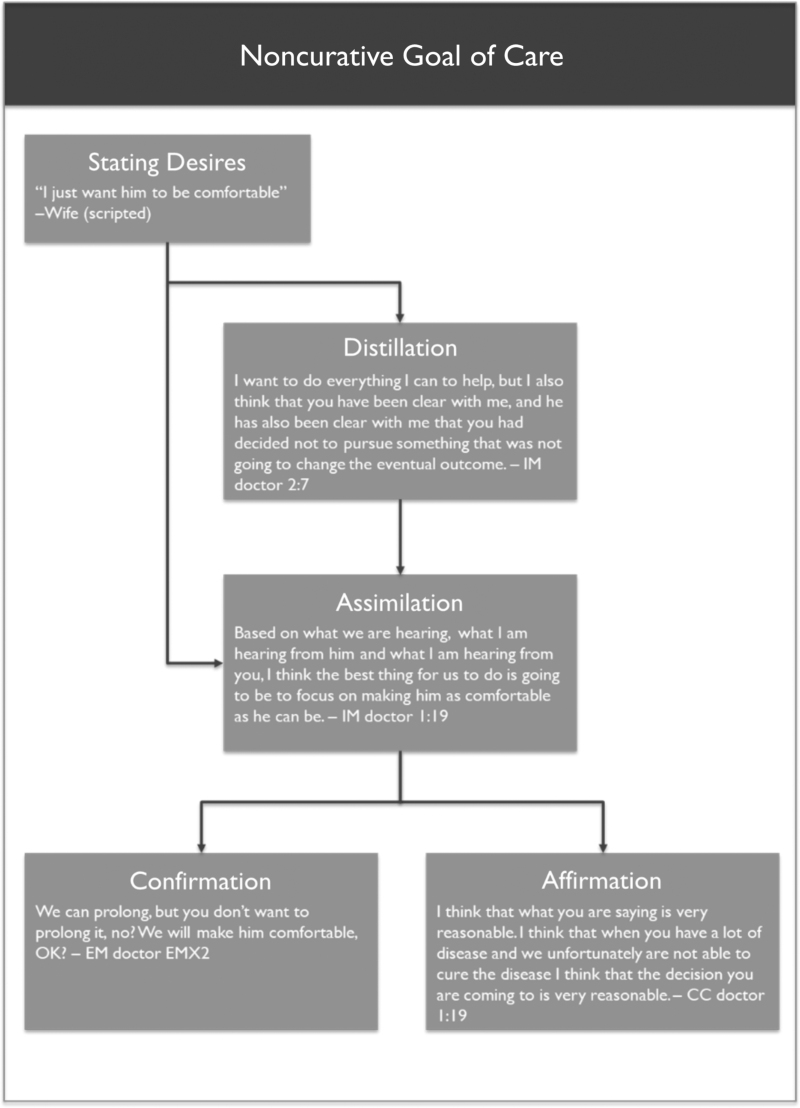
Paradigm of understanding noncurative goals of care shared across physician types. CC, critical care.

Typically, the goals of care conversation was set in motion after the physician inquired about the family's wishes regarding intubation or invasive management, and the patient or surrogate responded with “no tube” or “I want to keep him comfortable” (Fig. 2, Box A). Not yet a noncurative goal of care, these expressed desires opened the door for the physicians to begin discussing treatment goals. Some physicians took the opportunity to distill the expressed desires and to explicitly reframe the family's goals as noncurative (Box B). The next step in the paradigm was for the physician to state his or her own noncurative goals of care (Box C); nearly all of the physicians who explicitly named their own goals referred to the patient's expressed desires, but most of the physicians who had synthesized patient desires into patient goals also made statements to show that their physician goals were in alignment. At this point of the paradigm, physicians were not only describing the treatment being offered, but they were also aligning the reasoning behind their treatments with the stated goals of the family. After naming their goals, some physicians proceeded to confirm their statements (Box D) with the family (“this is what I've heard you say which is causing me to treat for comfort—do you agree with this plan?”). Finally, a small subset of physicians chose to affirm the family in their decision (Box E) by making statements such as “I would do the same” or “I understand why you are making this decision,” statements that normalized the family's goals and, ostensibly, helped the physician identify with the family.

#### Specialty comparison

In general, practitioners of all specialties whose interactions contained the code “noncurative goals of care” demonstrated at least one aspect of the aforementioned conversational paradigm (Fig. 2). As shown in the quantitative analysis, IM physicians were more likely than CC physicians to have the code for “noncurative goals of care” appear in their interactions with the patients. IM physicians, tended to utilize multiple components of the conversational paradigm, or to circle back to stated goals of care when confirming different aspects of the treatment plan. CC physicians, however, were the main contributors to the final aspect of the paradigm (Box E), in which the physician named and affirmed the family's goals of care.

Other physicians, who did not fit neatly into the noncurative paradigm as outlined earlier, still included some components of it in their interactions with the patient, but omitted other aspects, potentially to the detriment of the shared decision-making process. Many physicians for whom the noncurative code was absent did elicit the patient's desires and made treatment decisions based on the desires without ever explicitly defining the goals of care. These physicians' inferences about the patient's goals of care were correct, but the physicians in this group neither verbally synthesized the family's statements (Box B) nor outlined their own thought processes, thereby missing the opportunity to confirm that the physician and the patient understood one another (Boxes C and D). The few cases in which interactions included patient goals but not physician goals were marked by the physician summarizing patient wishes (Box B) and then treating accordingly, but never assimilating the patient's goals to the physician's goals to show how those aligned (Box C).

### Oblique references to death (K41)

#### Description of code

“Oblique references to death” were coded when a speaker invoked the concept of end of life without using the word “die” or any of its derivatives. These indirect references to death arose in three major contexts: intentional avoidance of the word “die,” testing the waters before a direct reference, and physician discomfort with discussing death. To clarify, the initial coding examined both oblique references to death (K41) and direct references to death (K40); only the former was statistically different between physician groups.

#### Stylistic avoidance

We acknowledge that in a review of transcribed encounters it is impossible to definitively assign intention to word choice. However, many interactions contained a seemingly studious avoidance of the word “die” although conveying the same meaning. It is possible that these physicians assumed that patients or families do not want to hear the word “death,” but felt that it was important for the family to know the prognosis. Statements such as “He may be approaching the end of his life” (Participant 2–15), although coded as oblique, certainly convey the same semantic function as a direct reference to death. Many encounters that contained no direct references to death nevertheless contained similarly strong language that was intended to convey the same meaning, and seems to indicate that some physicians were interested in conveying a sense of impending mortality without using the word “death”: “Mr. Jenkins do you understand that you might pass away tonight?” (Participant 1–19).

#### Testing the waters

Many encounters showed a gradient of obliqueness, in which the concept of dying was developed over the course of the discussion. These physicians often exhibited a preference for “testing the waters,” using an oblique reference to death to assess patient and family receptiveness and becoming more pointed in their language as the conversation proceeded. In these encounters, physicians usually opened with an oblique reference to death, a “shot across the bow” in which they expressed their concern that it may be “Howard's time” (Participant 1–1) or that they noticed that he is “really sick” (Participant 1–21). These openers allowed the family to press for more information, at which point the physician would more openly state his or her concern that the patient was dying. Some of these physicians proceeded to make a direct reference to death, whereas others made oblique but pointed statements, as discussed earlier. The following conversation actually contains a direct reference to death, but is an instance of a physician pulling aside the patient's wife to get a better sense of the patient's readiness to discuss end of life:

[ASIDE, WHISPERED DISCUSSION HELD]

Participant 1–21: *Ms. Jenkins, can you step to the side with me?*

Surrogate: *Sure*.

Participant 1–21: *So I know this must be very hard for you but I think he's really sick. I think he's going to die soon. [INAUDIBLE] I was afraid, and that's why I didn't want to say it in front of him. Do you think he would want me to talk to him directly about this?*

#### Physician discomfort

A small set (3 IM and 1 EM) of discussions highlighted a physician's personal discomfort discussing the death of his or her patient. The most apparent instances contained a paradigm in which the physician would offer a vague or oblique statement about a patient's prognosis, the patient's family would ask for clarification, and the physician would continue to obfuscate. Physicians who fell into this category typically relied on discussion of vital signs, would begin conversations about prognosis but break them off to confirm treatment plans or elements of the patient's history, or began discussion of comfort treatments without explicitly explaining why comfort was being pursued:

Surrogate: *Yeah, right. Uh huh, but I don't know what kind of end-of-life… I don't know what you're talking about.*

Participant 2–11: *Yeah, I'll be more clear here. If you can hold on a second, I'll talk to you a little bit more. Any help with the blood pressure medications at all with the vasopressors?*

Nurse: *No change.*

Participant 2–11: *Okay. Are we maxed out on the Levophed or can we keep going up on it until…*

Participant 2–11: *So, what I'm trying to say is that I'm a little concerned that where we're at right now is that his symptoms have… We're working really hard to try to treat the infection. The problem with infections is it can take a while for antibiotics to work.*

#### Specialty comparison

The quantitative data showed that IM physicians and CC physicians used oblique references to death more often than EM physicians (count data), and that IM doctors had oblique references to death present in more conversations than EM doctors (binary data). Physicians from all three categories tended to show some sensitivity to the word “death.” Most physicians began by assessing patient readiness before committing to a full goal of care conversation, often leading with a reference to prognosis or an indirect reference to death. Physicians from all three specialties were also represented in the “intentional avoidance” usage category. They effectively and repeatedly conveyed the concept of death without explicitly using the word. The final category of usage that disguised physician discomfort was infrequent; however, IM physicians were the most frequently involved in these conversations, with one EM provider also exhibiting significant discomfort.

## Discussion

This mixed-methods analysis of simulated end-of-life discussions demonstrates significant variability across different physician specialties with regard to their approach to end-of-life discussions. In particular, IM physicians were more likely than the other physician types to discuss noncurative goals of care, reference death obliquely, combine “comfort” with an oblique reference to death, elicit patient noncurative goals of care, and offer their own goal of care as noncurative. We also found that IM-trained doctors are more frequently directive with regard to preferences, values, and goals of care.

We propose that these differences arose in response to the settings of practice in which these different specialties operate. Prudent care dictates that practitioners use the right means to achieve the right end of patient-centered care; variations in the characteristics and demands of the diverging practice settings should lead to variations in the actions that can be considered prudent. Hospital-based IM physicians take care of patients who can be quite sick, but the setting is less acute than the emergency department or CC ward.^27^ In the typical hospital setting, physicians and patients may have more time for optimal shared decision making, and patients may be more likely to be at or near their mental baseline for these conversations. In addition, IM hospitalists may expect to care for these patients over the course of several days,^28^ which increases their drive to build and maintain a therapeutic relationship.^29^ For these reasons, we suggest that IM physicians demonstrated the greatest complexity to their discussion of comfort and were unique in introducing the concept of temporal collaboration during these discussions. The IM tendency to present the inherent risks in comfort care may also stem from the emphasis and increased opportunity for informed consent present in the floor setting.^29^ The concept of relationship building and the less acute setting may also have contributed to the more complete use of the noncurative goal of care paradigm discussed earlier (Fig. 2); IM providers are most likely to have a longitudinal relationship with their patients, and so the increased social referencing in reminding patients of their stated preferences could be a signal for relationship building and maintenance. Conversely, the decreased acuity of care relative to the other, highly acute fields may have increased IM physician discomfort with discussing death, although it is again worth noting that this was present in a small minority of the transcripts.

EM physicians work in a fast-paced environment in which stabilization and disposition are emphasized.^30^ These physicians especially made use of “comfort” as a treatment plan, used less as an opening to a conversation about the meaning of comfort care and more as a treatment modality. Although this practice is rarely prudent in a more protracted care setting, it may be adaptive for the setting of emergency care—triage and referral are central to the practice, so it is important to place patients in treatment stratifications.^31^ The long-term nuances of comfort care are not managed by EM physicians, which is likely why there were fewer extended discussions of the components of care, fewer references to the relational aspects of comfort, and no talk of the temporal aspect of comfort care. These physicians also tended to use a more abbreviated noncurative goal paradigm, generally opting for a more efficient, less comprehensive path from patient desires to treatment decision. The EM physicians were fairly neutral in their discussion of death; there was little evidence of discomfort in the dataset, and EM physicians who utilized oblique references to death typically assessed the family's readiness to discuss prognosis.

CC physicians also practice in a high-acuity setting, although they often work with patients for a longer time course than EM providers. In this setting, the patient has already been labeled as high risk, and physicians must be vigilant to changes in a patient's course throughout the day. These physicians also frequently work with sedated or unconscious patients and, therefore, must be facile not only in caring for the patient directly but also in communicating with surrogates/families.^32^ This probably contributed to their references to relational aspects of comfort, and may have been especially relevant to their handling of noncurative goal of care conversations. CC providers most frequently incorporated their personal approval into the paradigm; perhaps these physicians are especially aware of the long-term mental wellness of families after the death of a loved one and thus recognize the healing effects of approbation in end-of-life experiences.^33^ Awareness of the family needs may also have contributed to the CC physicians' tendencies to test the waters before diving into discussion of prognosis.

Previous research has demonstrated differences among specialties in treatment of diseases and in communication about patient care.^34,35^ These findings add to the limited research in inter-specialty differences during end-of-life care, allowing us to explore the communication patterns of three specialties that frequently encounter terminal patients. Although specific best practices undoubtedly hold true across specialties, we may also suppose that certain differences in conducting end-of-life discussions are actually adaptations to the specifics of each specialty's practice setting.

Although this analysis is exploratory, the findings suggest that one potential intervention for improving EOL care would be to develop tools to help CC and EM physicians become more comfortable with referencing death, addressing noncurative goals of care, and making a clear recommendation that aligns patient care with elicited values. Interventions along these lines already exist,^6,36^ but our data provide additional evidence for their need. Furthermore, simulated clinical encounters such as the ones used in this study could be evaluated with our coding scheme as one way to assess the impact of interventions aimed at increasing physician skill in referencing death and noncurative goals of care.

Our findings also suggest an opportunity for a practice-specific framework for graduate medical education, especially in primary palliative care initiatives that take into account the different specialties' unique context, needs, and practices. The goal of identifying differences in practice should not necessarily be to eliminate them, but instead to identify which changes arose as a primary response to the environment of practice and which changes are an unanticipated (and potentially unhelpful) consequence of that practice. Taking EM as an example, the general trend toward fewer relational uses of the concepts of comfort and noncure is probably adaptive given the volume of patients in the department and the reality that most patients with serious illnesses will in fact be admitted to another team for long-term management of these conditions. In contrast, trends toward less aptitude in identifying patient goals of care are more concerning and should be a target for intervention, since early differentiation to the comfort-focused treatment paradigm could have better patient and family outcomes.^9^

In addition to the contributions this study makes to understanding specialty-specific differences in these simulated end-of-life conversations, we believe this study advances a valuable method for exploring complex behaviors, including but not limited to the exhibition of virtues, during clinical care. The methodology of this study is unique in that actions reflective of the virtue of prudence were identified *a priori*, refined through iterative exploration of the sample of patient encounters, quantified, then revisited through qualitative analysis. We contend that this method, although complex, delivers a rich understanding of both the use and context of a complex behavior (prudence) and can set the foundation for study of other complex behaviors that play a role in health care, such as emotional responsiveness, integrity, or respect.

This study has several limitations. First, the setting was a simulation rather than an actual patient encounter. This allowed for a consistent clinical scenario with well-established patient wishes, but physicians may have felt more comfortable making the lower-stakes end-of-life decisions in a simulation than they would have with a truly dying patient. In addition, with the knowledge that their behavior was observed, physicians may have behaved differently, creating a Hawthorne effect. Physicians were a convenience sample of volunteers who agreed to take part in a study of end-of-life care. Physicians were recruited across multiple institutions and represented a diversity of experience in practice, but there is likely an inherent bias in a sample of physicians who would agree to participate in such a study. In addition, all participants were recruited from three academic institutions with well-established palliative care programs, which could either increase physician end-of-life skills through access to specialty training or decrease these skills due to the presence of expert consultants in difficult situations. Finally, although we believe that our methodology provides a beneficial approach to the rigorous study of complex behavior, we allow that there is an element of circularity inherent in defining prudence, measuring its presence qualitatively, and confirming and expanding on our results with the subsequent thematic analysis. In this way, the findings of the quantitative analysis informed the final qualitative analysis rather than two independent analyses reaching the same conclusion. Ultimately, we do not believe this to be a limitation, but a confirmation that our iterative approach allows for a richer understanding of a nuanced behavior.

These studies are an intriguing first step into the exploration of interspecialty variability in goals of care conversations at the end of life. Further study could focus on the evaluation of other specialty groups, both to learn additional techniques and tendencies and to influence the training specific to these trainees. Based on the findings of these studies, it may be important for medical educators to develop targeted programs of training that match best practices in goal of care discussions with the settings where these practices are best suited.

## Supplementary Material

Supplemental data

## Abbreviations Used

ANOVA: analysis of variance
CC: critical care
CMO: comfort measures only
DNR/DNI: do not resuscitate/do no intubate
EM: emergency medicine
ICU: intensive care unit
IM: internal medicine
KW: Kruskal–Wallis
ND: no difference

## Notes

^*^ Virtue theory traces roots to Plato and Aristotle. It was the primary approach to moral philosophy in the West until the Enlightenment, and it has enjoyed a renaissance in in the past 50 years because it addresses some the limitations exposed by “post-modern” critics of moral philosophy. Although “virtue” or “virtuous” can be used in common parlance to indicate normative “goodness,” our use of the term is technical and denotes a trait of character—a well-entrenched disposition that goes beyond mere habit such that possessing “a virtue is to be a certain kind of person with a certain sort of complex mindset” (https://plato.stanford.edu/entries/ethics-virtue). An honest person is the kind of person who tells the truth and does not lie. A courageous person is the kind of person who acts rightly even in the face of danger, neither rash nor cowardly. A prudent person is the kind of person who, by and larger, achieves the right end through the right means. These traits of character or well-entrenched dispositions are not necessarily normatively good (e.g., an individual's character can be dishonest, cowardly, rash, or otherwise vicious). And different people's character may be more or less reliably virtuous (or vicious). As such, virtue ethics focus on character rather than rules (deontology, e.g., Kant) or consequences (consequentialism, e.g., J.S. Mill). One critical implication of this focus on character rather than rules or consequences is the recognition that virtues are transmitted through particular communities of practice that embody the virtues necessary for those particular practices. The prudent virtue of a mason knows how to mix mortar and stack bricks so that a wall stands straight and bears weight. Physicians have a different kind of prudence than masons, and we hypothesized that distinctions between specific communities of physician practice would shape the form of prudence transmitted within those specific communities of physician practice. Our challenge was to demonstrate empirical differences in behavior that could be explained by this understanding of virtue and the formation of character through communities of practice.
