# Supplementary material for: Cultures of Practice: Specialty-Specific Differences in End-of-Life Conversations
Source: Palliat Med Rep. 2021 Mar 24;2(1):71–83. doi: 10.1089/pmr.2020.0054 (PMC8043084; doi:10.1089/pmr.2020.0054)
Supplement: Supplemental data [file Supp_Data.docx]

*Participant Characteristics Stratified by Practice Setting*

|  | | **Emergency**  **(N=23)** | **Intensivist**  **(N=36)** | **Hospitalist**  **(N=29)** |
| --- | --- | --- | --- | --- |
| **Characteristic** | | Mean or Frequency | Mean or Frequency | Mean or Frequency |
| Age (years) |  | 46.2 (SD: 11.8) | 38.9 (SD: 6.5) | 34.2 (SD: 4.7) |
| Sex | Male | 16 (69.6%) | 29 (80.6%) | 20 (69.0%) |
|  | Female | 7 (30.4%) | 7 (19.4%) | 9 (31.0%) |
| Race | White | 17 (73.9%) | 21 (58.3%) | 14 (48.3%) |
|  | Hispanic | 1 (4.4%) | 1 (2.8%) | 1 (3.5%) |
|  | Asian | 5 (21.7%) | 12 (33.3%) | 13 (44.8%) |
|  | Black | 0 (0%) | 1 (2.8%) | 1 (3.5%) |
|  | Unknown | 0 (0%) | 1 (2.8%) | 0 (0%) |
| Years since Medical School Graduation |  | 18.7 (SD: 12.0) | 11.0 (SD: 6.7) | 7.8 (SD: 4.4) |
